# Supplementary material for: Divergent global-scale temperature effects from identical aerosols emitted in different regions
Source: Nat Commun. 2018 Aug 17;9:3289. doi: 10.1038/s41467-018-05838-6 (PMC6097985; doi:10.1038/s41467-018-05838-6)
Supplement: Supplementary file 1 — Supplementary Information [file 41467_2018_5838_MOESM1_ESM.pdf]

Supplement to

Divergent Global-Scale Temperature Effects from Identical Aerosols Emitted in Different Regions

Persad and Caldeira

Supplementary Figures

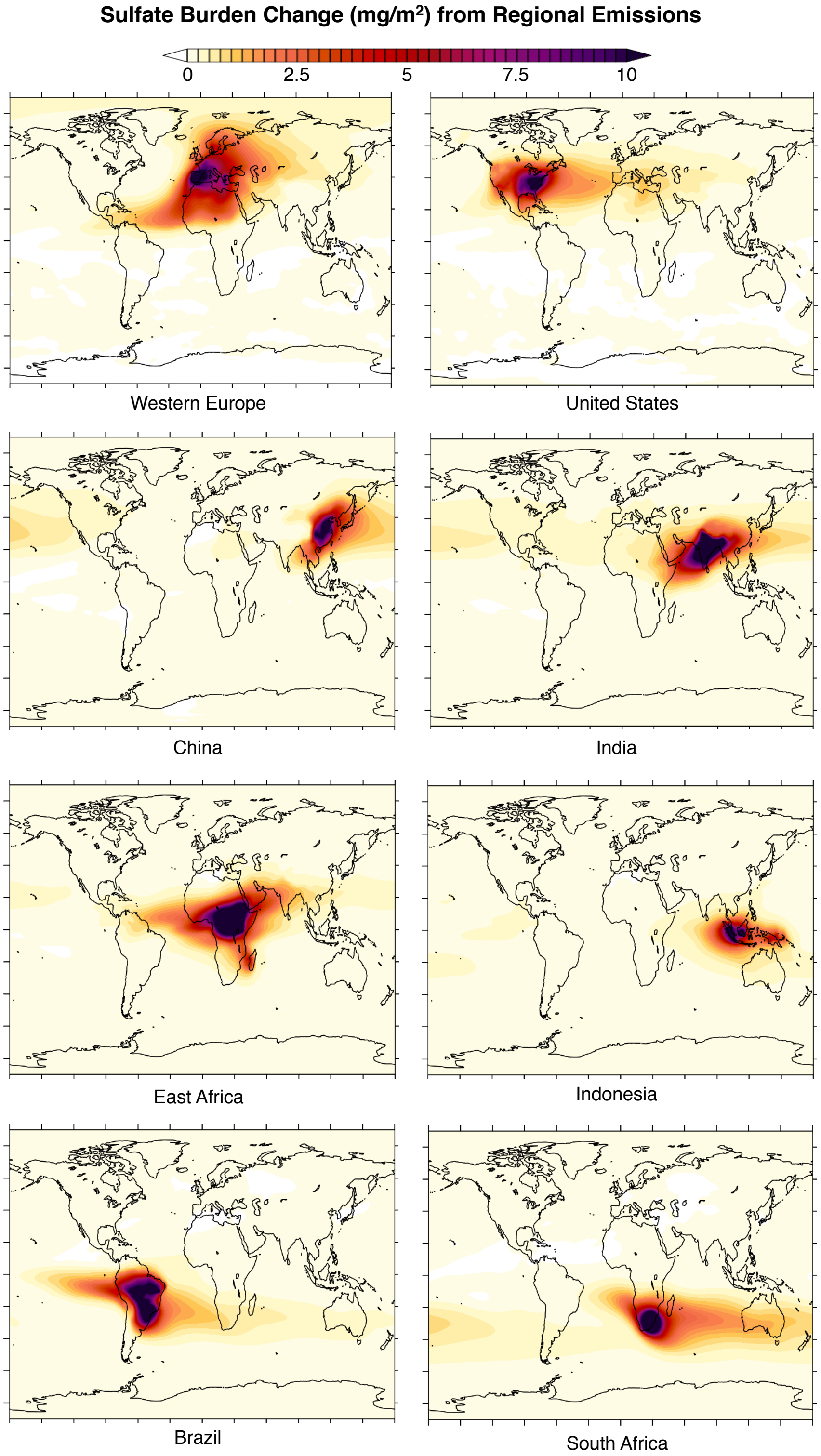

Supplementary Figure 1. The change in total atmospheric burden of sulfate aerosol (mg/m^2) from 8 emissions regions indicates that atmospheric burdens remain relatively concentrated near the emissions region, particularly in the meridional direction. Changes at all grid points are statistically significant at the 95% confidence level.

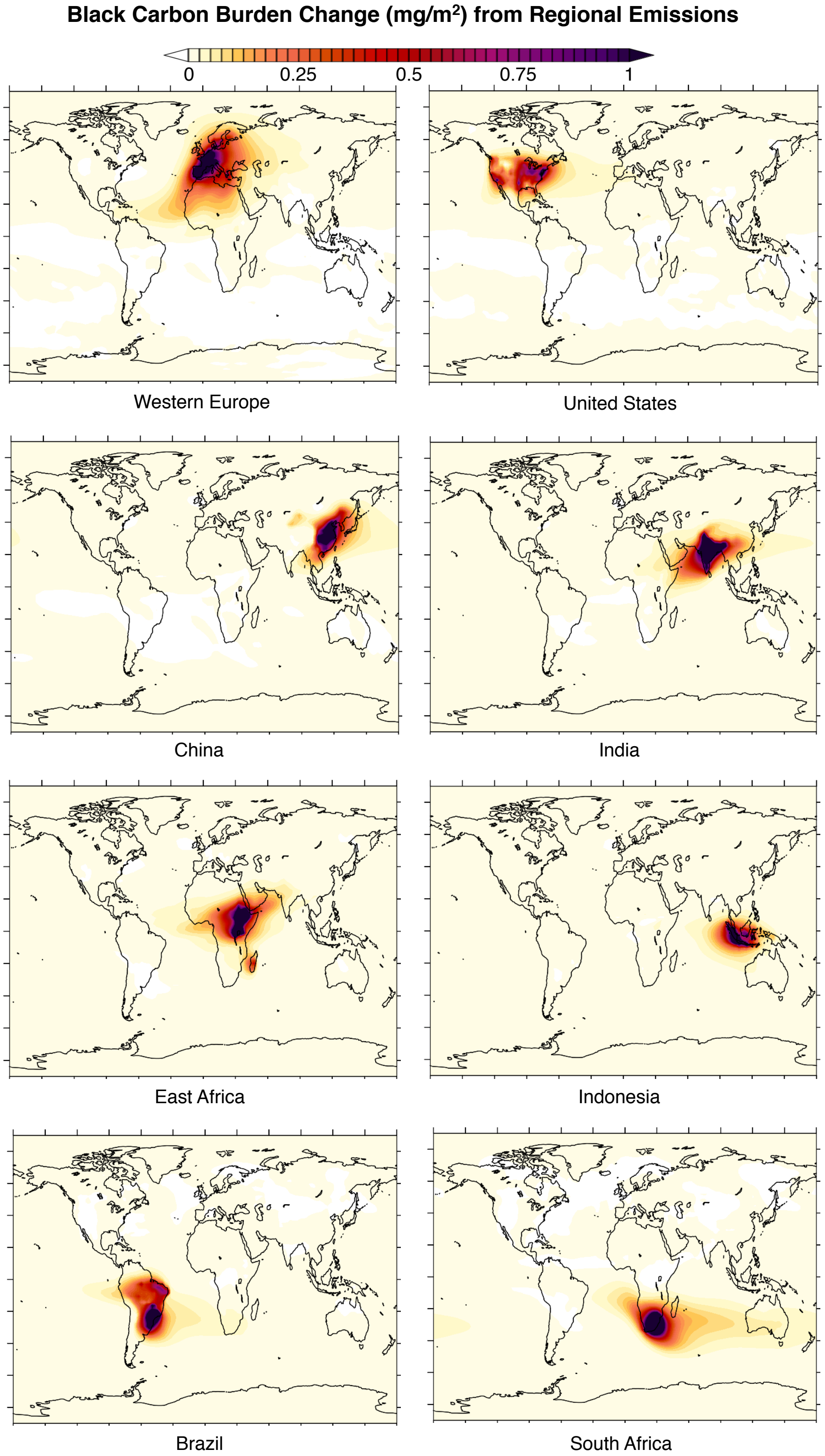

Supplementary Figure 2. The change in total atmospheric burden of black carbon aerosol (mg/m^2) from 8 emissions regions indicates that atmospheric burdens remain relatively concentrated near the emissions region, particularly in the meridional direction. Changes at all grid points are statistically significant at the 95% confidence level.

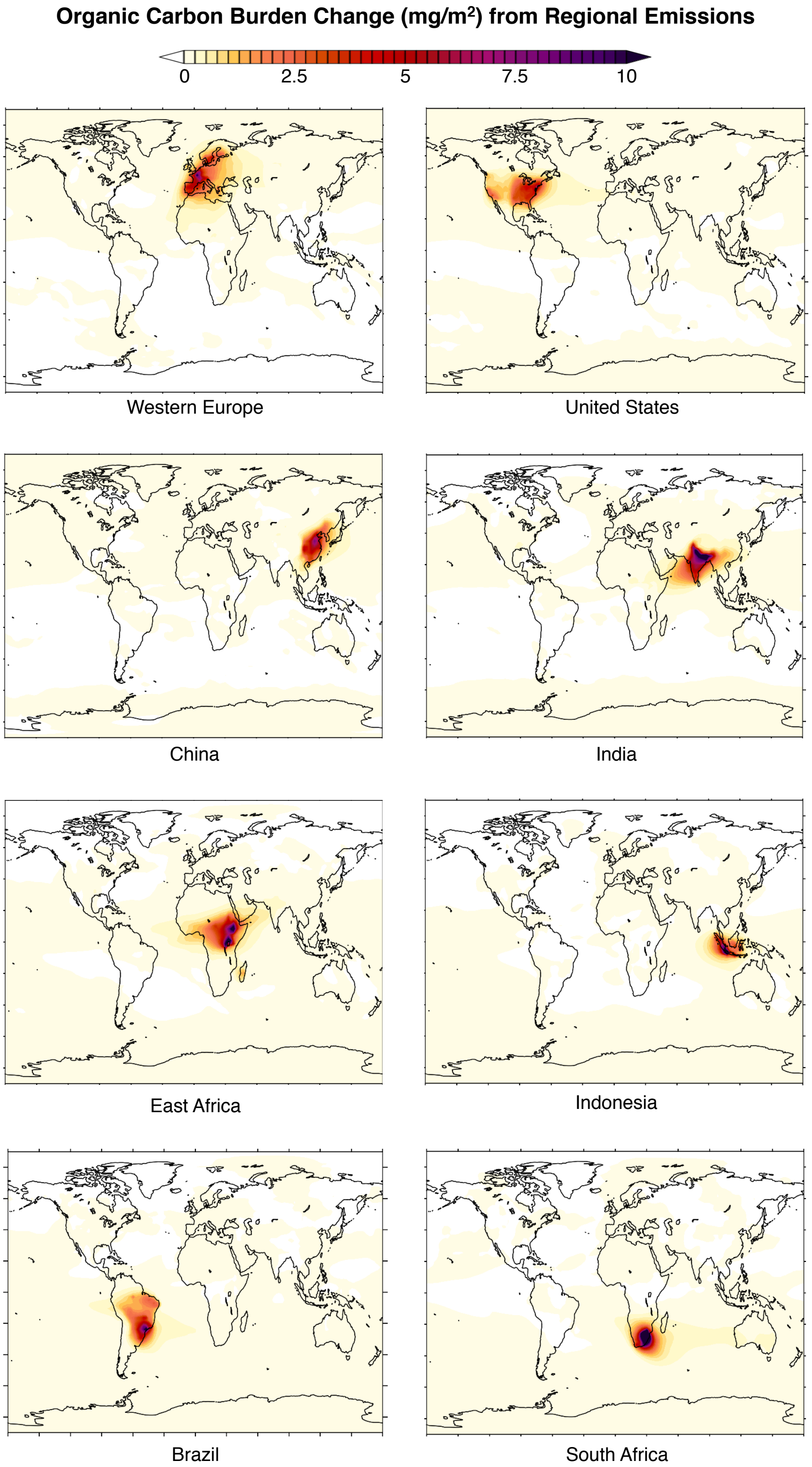

Supplementary Figure 3. The change in total atmospheric burden of organic carbon aerosol (mg/m^2) from 8 emissions regions indicates that atmospheric burdens remain relatively concentrated near the emissions region, particularly in the meridional direction. Changes at all grid points are statistically significant at the 95% confidence level.

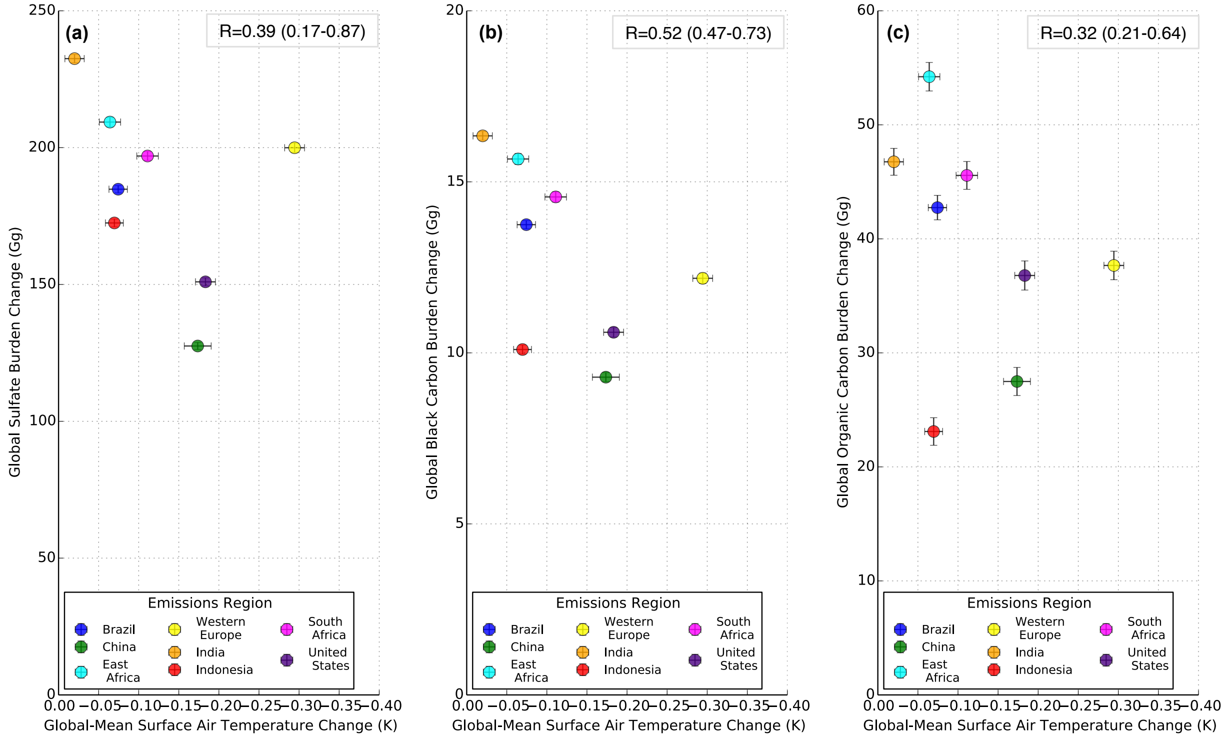

Supplementary Figure 4. Global total changes in the burden resulting from identical regional emissions of the 3 aerosol species—**(a)** sulfate aerosol, (**b)** black carbon aerosol, and **(c)** organic carbon aerosol—perturbed in this study are shown versus the global-mean surface air temperature resulting from that region's emissions. R values in the top right are given with ranges established via jackknife resampling (see Materials and Methods), and show that the relative change in burdens of the individual species have limited power to explain variability in global-mean temperature change resulting from the regional emissions. Error bars capture the standard error and are the same size as markers for the vertical dimensions of (a) and (b).

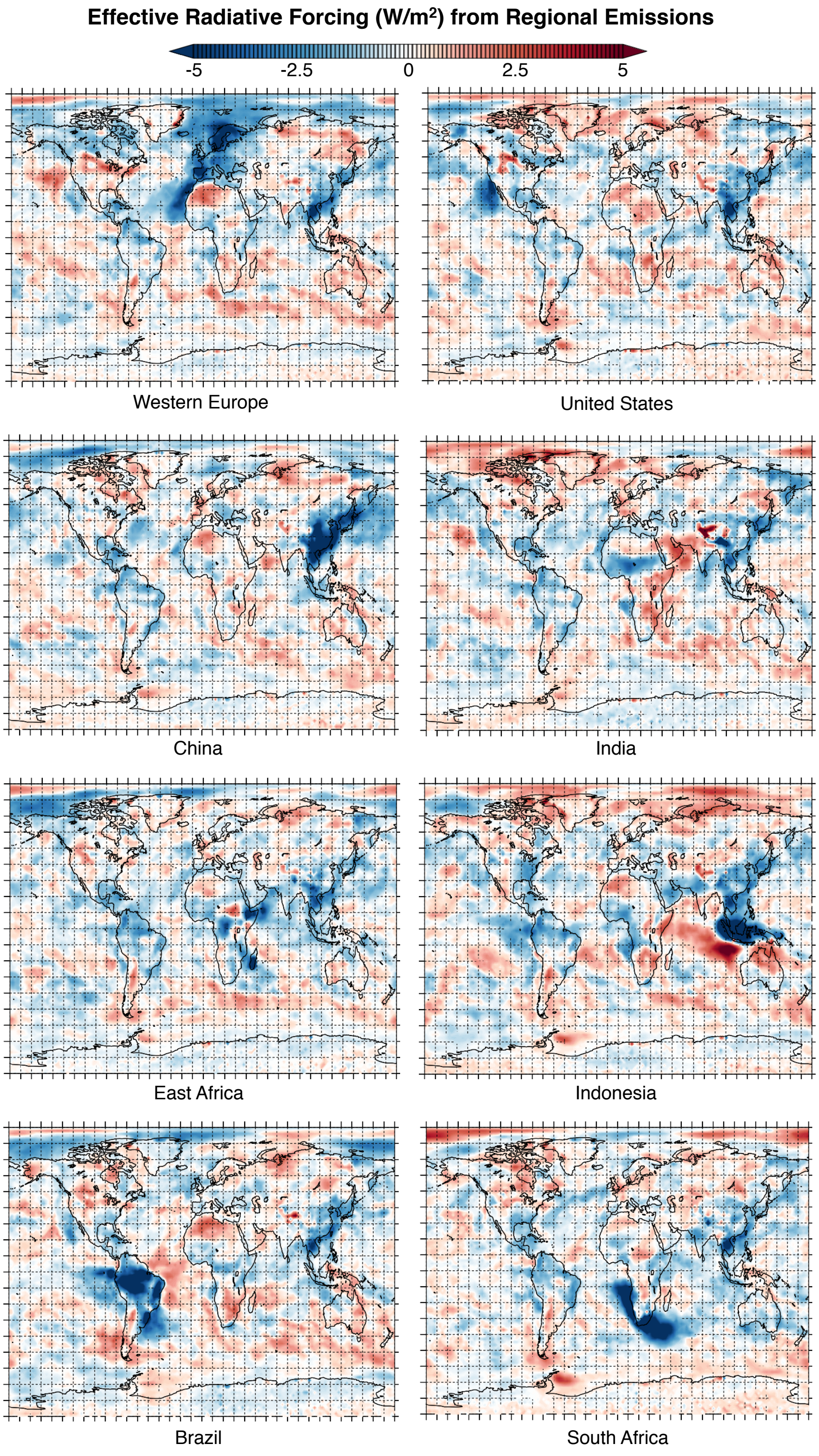

Supplementary Figure 5. Spatial patterns of top-of-atmosphere effective radiative forcing (ERF) resulting from identical emissions from 8 regions. Grid markings indicate regions that are not statistically significant at the 95% confidence level via t-test.

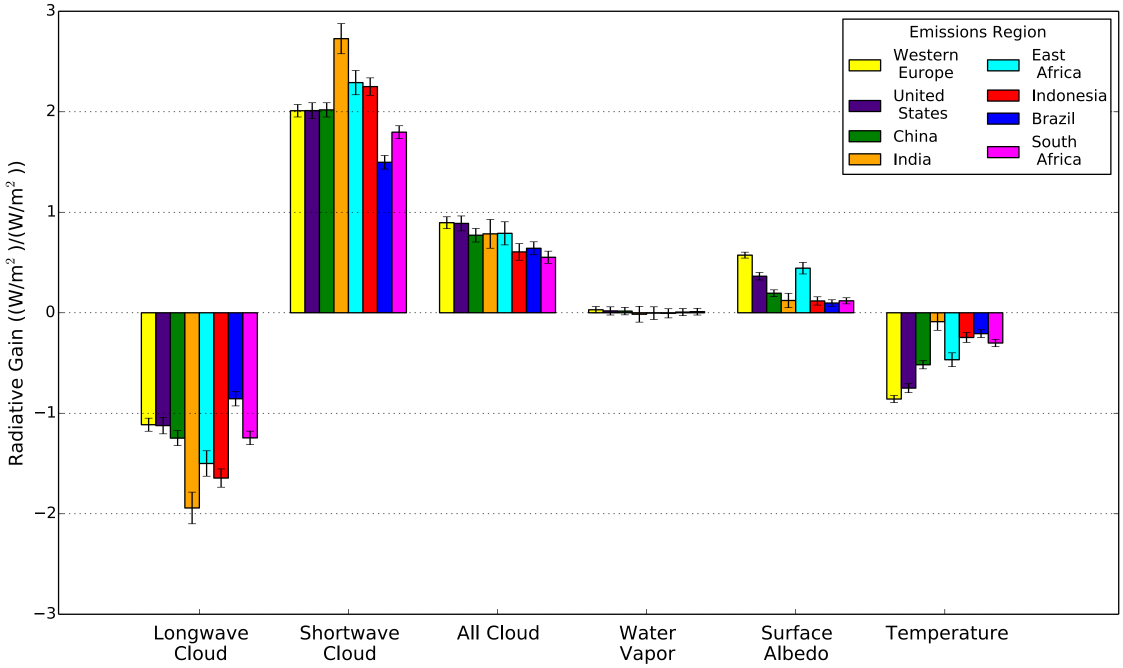

Supplementary Figure 6. The global-mean radiative gain from individual climate feedbacks – i.e. the additional top-of-atmosphere flux change from the feedback due to a unit of effective radiative forcing (Figure 2c) – generated by each emitting region. The temperature feedback includes both the lapse rate and Planck feedbacks. Error bars capture the standard error.

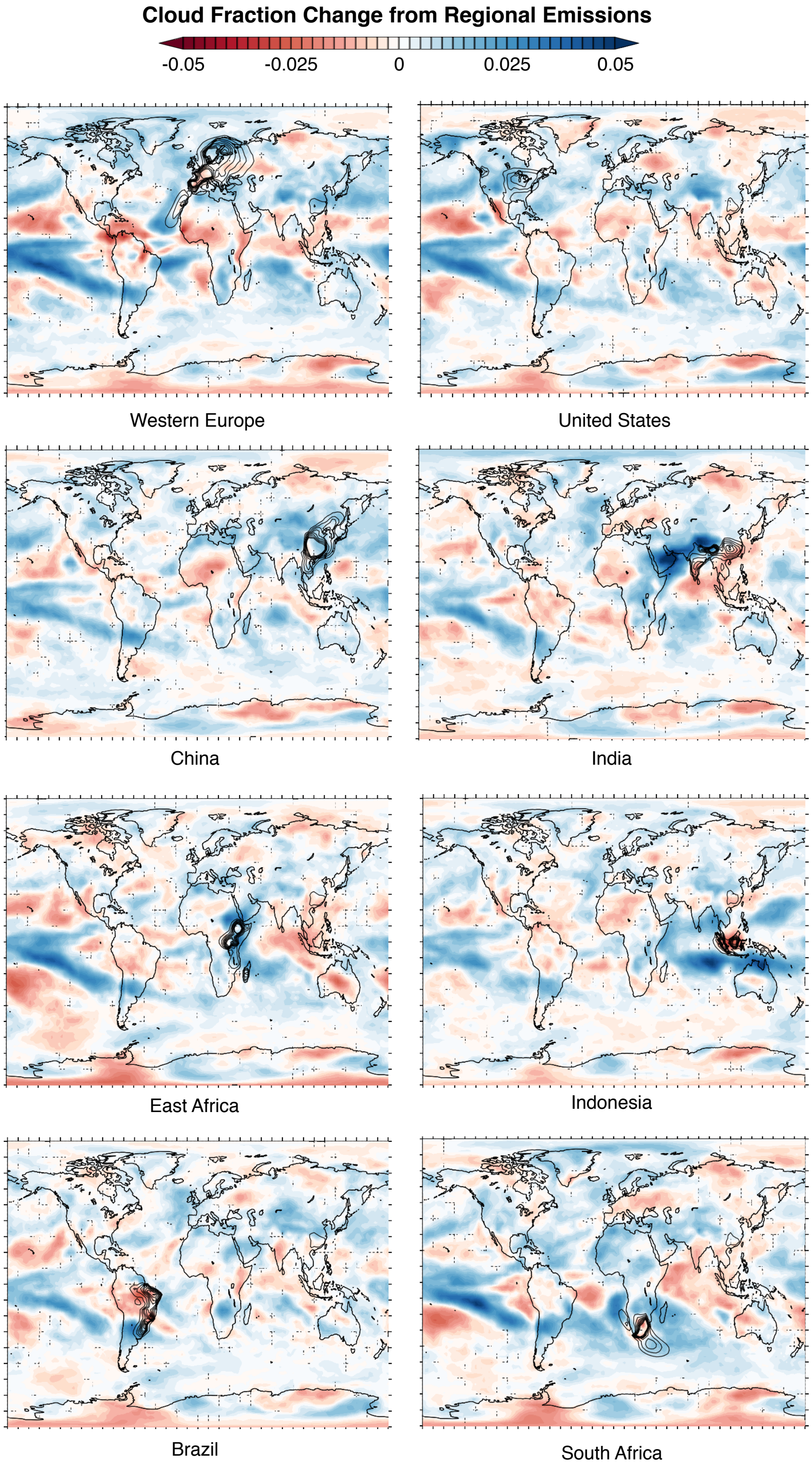

Supplementary Figure 7. The changes in cloud fraction (shading) and cloud droplet number concentration (contours) due to emissions from 8 regions. Contours are at intervals of 5 x 10^9 droplets/m^2. Grid markings indicate regions that are not statistically significant at the 95% confidence level via t-test.

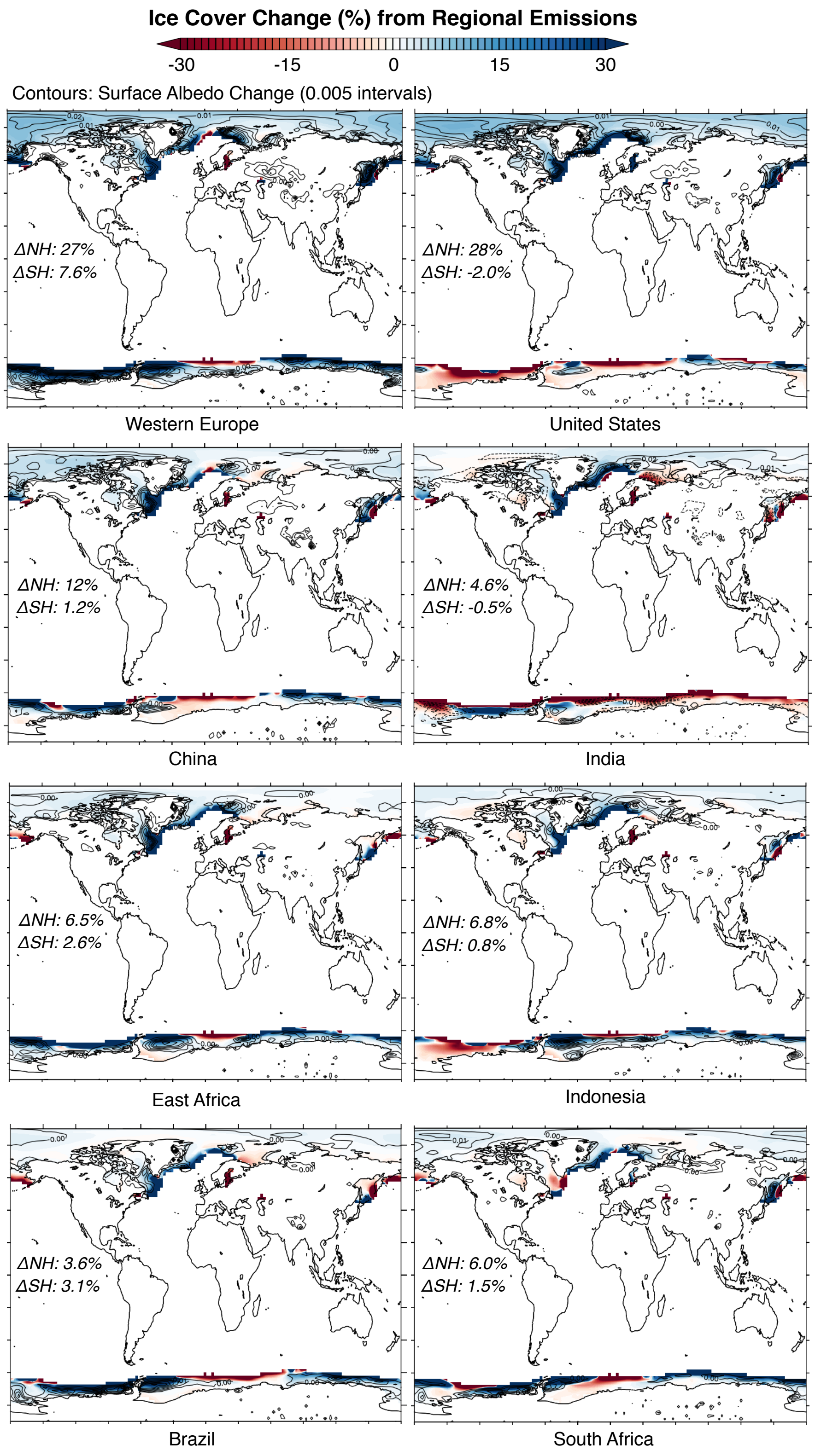

Supplementary Figure 8. The change in annual-mean ice cover (% change relative to climatology) due to emissions from 8 regions (shading) and in surface albedo (contours) indicate differing ice albedo feedbacks depending on emissions region. Changes averaged over the Northern Hemisphere (ΔNH) and Southern Hemisphere (ΔSH) are also given. Contours are at intervals of 0.005 in terms of change in fractional albedo.

Supplementary Tables

Table 1. **Summary of Data.** Data presented in Figures 1b, 2, and 4 and Supplementary Figure 5 are shown. Error ranges are given as the standard error (see Materials and Methods).

**Emitting Region**	**Brazil**	**China**	**East Africa**	**Western Europe**	**India**	**Indonesia**	**South Africa**	**United States**
**Global-Mean Surface Air Temperature Change (K)**	-0.074	-0.174	-0.064	-0.294	-0.020	-0.070	-0.111	-0.183
Global-Mean Surface Air Temperature Change Error (K)	*0.011*	*0.017*	*0.013*	*0.012*	*0.012*	*0.011*	*0.013*	*0.012*
**Regional-Mean Surface Air Temperature Change (K)**	-0.160	-0.487	-0.137	-0.505	-0.424	-0.137	-0.690	-0.449
Regional-Mean Surface Air Temperature Change Error (K)	*0.066*	*0.033*	*0.077*	*0.059*	*0.029*	*0.046*	*0.058*	*0.101*
**Global-Mean Effective Radiative Forcing (W/m^2)**	-0.190	-0.221	-0.093	-0.223	-0.085	-0.160	-0.210	-0.169
Global-Mean Effective Radiative Forcing Error (W/m^2)	*0.036*	*0.035*	*0.034*	*0.035*	*0.036*	*0.040*	*0.035*	*0.033*
**Efficacy ($K/W/m^2$)**	0.392	0.784	0.691	1.319	0.235	0.434	0.529	1.087
Efficacy Error ($K/W/m^2$)	*0.060*	*0.076*	*0.143*	*0.055*	*0.142*	*0.070*	*0.064*	*0.074*
**Black Carbon Column Burden (Gg)**	13.7	9.3	15.7	12.2	16.3	10.1	14.6	10.6
Black Carbon Column Burden Error (Gg)	*0.120*	*0.132*	*0.160*	*0.151*	*0.129*	*0.132*	*0.150*	*0.139*
**Sulfate Column Burden (Gg)**	184.8	127.5	209.3	200.0	232.5	172.5	196.9	151.0
Sulfate Column Burden Error (Gg)	*1.332*	*1.295*	*1.769*	*1.738*	*1.516*	*1.429*	*1.639*	*1.454*
**Organic Carbon Column Burden (Gg)**	42.7	27.5	54.2	37.7	46.8	23.1	45.6	36.8
Organic Carbon Column Burden Error (Gg)	*1.072*	*1.232*	*1.250*	*1.252*	*1.178*	*1.208*	*1.221*	*1.276*
